# St. Thomas Modified Cardioplegia Effects on Myoblasts’ Viability and Morphology

**DOI:** 10.3390/medicina58020280

**Published:** 2022-02-13

**Authors:** Rafał Nowicki, Katarzyna Bieżuńska-Kusiak, Julita Kulbacka, Anna Choromanska, Małgorzata Daczewska, Stanisław Potoczek, Maciej Rachwalik, Jolanta Saczko

**Affiliations:** 1 Department of Cardiac Surgery, Wroclaw Medical University, Curie-Skłodowskiej, 50-369 Wrocław, Poland; maciej.rachwalik@umw.edu.pl; 2 Department of Molecular and Cellular Biology, Wroclaw Medical University, Borowska 211A, 50-368 Wrocław, Poland; katarzyna.biezunska-kusiak@umw.edu.pl (K.B.-K.); julita.kulbacka@umw.edu.pl (J.K.); anna.choromanska@umw.edu.pl (A.C.); jolanta.saczko@umw.edu.pl (J.S.); 3 Department of Animal Developmental Biology, Institute of Experimental Biology, University of Wroclaw, Sienkiewicza 21 St., 50-335 Wrocław, Poland; malgorzata.daczewska@uwr.edu.pl; 4 Department of Hematology, Blood Neoplasms and Bone Marrow Transplantation, Wroclaw Medical University, Wybrzeże Pasteura 4, 50-367 Wrocław, Poland; stanislaw.potoczek@umw.edu.pl

**Keywords:** rat heart myocardium cells, proliferating antigens, thiol groups, cold crystalloid cardioplegia

## Abstract

*Background and Objectives*: The cardioplegic arrest of the heart during cardiosurgical procedures is the crucial element of a cardioprotection strategy. Numerous clinical trials compare different cardioplegic solutions and cardioprotective protocols, but a relatively small number of papers apply to in vitro conditions using cultured cells. This work aimed to analyze whether it is possible to use the rat heart myocardium cells as an in vitro model to study the protective properties of St. Thomas cardioplegia (ST2C). *Methods:* The rat heart myocardium cells-H9C2 were incubated with cold cardioplegia for up to 24 h. After incubation, we determined: viability, confluency, and cell size, the thiol groups’ level by modifying Ellman’s method, Ki67, and Proliferating Cell Nuclear Antigen expression (PCNA). The impact on cells’ morphology was visualized by the ultrastructural (TEM) study and holotomograpic 3D imaging. *Results:* The viability and confluency analysis demonstrated that the safest exposure to ST2C, should not exceed 4h. An increased expression of Ki67 antigen and PCNA was observed. TEM and 3D imaging studies revealed vacuolization after the longest period of exposure (24). *Conclusions:* According to obtained results, we conclude that STC can play a protective role in cardiac surgery during heart arrest.

## 1. Introduction

The cardioplegic arrest of the heart during cardiosurgical procedures is the crucial element of a cardioprotection strategy. However, the cessation of coronary arteries perfusion creates the risk of ischemic and reperfusion injury [1,2]. The main idea of the cardioplegia application is to assure protection, which is a safe, uncomplicated procedure.

The protective effect of cardioplegia is mainly based on potassium ions which impact the cellular membrane. The high serum concentration of potassium depolarizes the cell membrane suppressing the electric and mechanical activity of cardiomyocytes, causing cardiac arrest [3,4,5]. Cardioplegic solutions can induce cytotoxic effects because of the increased content of potassium ions or its anionic salts [6]; thus, this composition is still improved and developed. There are two types of cardioplegia used in the clinical practice based on the crystalloid solutions (e.g., Btertschneider’s formula or St. Thomas’ Hospital formula) and so-called blood cardioplegia (one of its components is the patient’s blood). There are also newly developed polarizing solutions containing adenosine, lidocaine, and magnesium (ALM), which also can protect coronary vasculature and enhance an inflammatory response [6]. Cardioplegia is administered to the coronary vessels just after the cross-clamping of the aorta. At that moment, the function of the heart and lungs is provided by the extracorporeal circulation (heart-lung machine). There are different ways of administration, various protocols, and physical conditions of the cardioplegic solution application. Although millions of open-heart operations have been made so far, the amount of cardioprotective technics proves its limitations, especially in the case of long cross-clamp (i.e., more than 4 h) [7,8,9]. The various clinical trials evaluate different cardioplegic solutions and cardioprotective procedures, but only a few papers apply to in vitro conditions with the use of cultured cells [4,5,10,11,12].

This work aimed to analyze the possible use and the cellular effects of St. Thomas 2 based cardioplegia (ST2C) in the rat heart myocardium cells as an in vitro sensitive model. The idea of the study was to estimate the safe time exposure of cardiac cells, which could be useful in heart protection and new cardioplegic solutions in cardiac surgery procedures.

## 2. Materials and Methods

### 2.1. Cell Culture

Rat heart myocardium cells-H9C2 (ATCC^®®^) were used in the study. The cell line was grown in DMEM (Lonza, Basel, Switzerland) medium with the addition of 10% fetal bovine serum (FBS, Lonza, Basel, Switzerland), 2 mM Glutamine, and 100× penicillin/streptomycin (Sigma-Aldrich, Merck Life Science, Poznan, Poland). For experiments, cells were removed by trypsinizing (0.25% Trypsin-EDTA solution, Sigma-Aldrich, Merck Life Science, Poznan, Poland) and washed with PBS. The cells were maintained in a humidified atmosphere at 37 °C and 5% CO_2_. The medium was exchanged 2–3 times per week.

### 2.2. Culturing with Cold Cardioplegia

Crystalloid cardioplegia based on St. Thomas no. 2 cardioplegia (ST2C) [13] was prepared in the Department of Cardiac Surgery, Wroclaw Medical University (Poland). The following composition was prepared: 50 mL of Ringer solution (Fresenius Kabi, Warsaw, Poland), 5 mL of KCl (2.5 mEq/mL), 20 mL of 20% magnesium sulfate (Polpharma, Poland), and 25 mL of 20% mannitol (Baxter, Warsaw, Poland). Rat heart myocardium cells were incubated for up to 24 h in the modified cardioplegia solution.

### 2.3. Cell Viability

Cell viability was assessed by MTT assay (3-(4,5-dimethylthiazol-2-yl)-2,5-diphenyltetrazolium bromide, Millipore, Poznan, Poland), which is based on the detection of the mitochondrial activity. First, cells were trypsynized and seeded on 96-well plate at density 10^4^ cells/well. After 24 h, the exposure with ST2C solution was initiated during 1, 2, 12, and 24 h. Post the exposition MTT assay was performed according to the manufacturer protocol, and formazan crystals were dissolved in acidified isopropanol. The absorbance values were measured at 570 nm by the multiplate reader (Glomax, Promega, GmbH, Mannheim, Germany). All the results were calculated as the percentage of viable cells in the relation to untreated control cells.

### 2.4. Cells’ Confluency and Volume

H9C2 ells were trypsynized and seeded on 96-well plate at density 104 cells/well. After 24 h, exposure with ST2C solution was initiated, and, simultaneously, an analysis of cells confluency and cell size was initiated every 1 h during 24 h. The measurements were performed on the live cell imaging platform CELLCYTE X (Sygnis, Warsaw, Poland) placed in the cell culture incubator to assure 37 °C and 5% CO_2_. High contrast Enhanced Contour mode was used to help distinguish detected structures. The results were collected by Cellink Studio software (Sygnis, Warsaw, Poland)

### 2.5. Holographic Tomography Microscopy (HTM)

Holographic tomography was performed using a 3D Cell Explorer microscope (Nanolive SA, Sygnis, Warsaw, Poland). Cardiomyocytes were seeded on cover microscopic glasses, and then exposed to ST2C solution for 1, 2 and 24 h. Following the incubation with cardioplegia solution, H9C2 cells were fixed in 4% paraformaldehyde, mounted in FluorshieldTM with DAPI (4,6-diamidino-2-phenylindole, Roth, Nuremberg, Germany) for nuclei visualization. Samples were placed on the basic microscopic slides and detected in Nanolive microscopy. Photographs of the samples were converted in Steve software (Sygnis, Warsaw, Poland).

### 2.6. Transmission Electron Microscopy (TEM)

The TEM studies were conducted after exposure of H9C2 cells to cold crystalloid cardioplegia for 1, 2, and 24 h. The cells were fixed in cold 2.5% glutaraldehyde in 0.1 mol/L cacodylate buffer (pH 7.3), postfixed in 1% OsO_4_, dehydrated, and embedded in Epon (POCH, Warsaw, Poland). Ultrathin sections were mounted on copper grids, stained with uranyl acetate and lead citrate, and examined under a Jem-100C (JEOL, Tokyo, Japan) electron microscope. The electron micrographs were taken systematically at ×5000 magnification.

### 2.7. Thiol Groups Level

Proteins damage was based on Ellman’s method. This method uses a reaction of 5,5’-dithiobis-2-nitrobenzoic acid (DTNB acid, Fluka, Munich, Germany) with thiol groups of proteins. Firstly, cells were exposed for 24 h to ST2C based cardioplegia in cell culture flasks (25 m^2^). After this period of time, cells were trypsinized and dedicated for thiol groups measurements. The level of –SH groups was measured spectrophotometrically on the basis of the absorbance at the wavelength of 412 nm. All measurements were performed on UV/Vis spectrophotometer (Jasco V-530, Medson, Paczkowo, Poland). Experiments were performed in triplicate.

### 2.8. Immunocytochemistry

Immunocytochemistry was performed using an ABC method. Briefly, cultures were fixed and dehydrated using 4% paraformaldehyde and an ethanol gradient, respectively. Samples were then permeabilized and blocked by incubation with 0.1% Triton X-100 (Sigma) in PBS. Expression of proteins was visualized with the mouse monoclonal antibody (1:100, Ki67, Santa Cruz; anti-PCNA; Proliferating Cell Nuclear Antigen PC10, DAKO). For conventional bright-field microscopy (peroxidase-ABC labeling), the samples were incubated with the diaminobenzidine-H_2_O_2_ mixture (DAKO) to visualize the peroxidase label, counterstained with hematoxylin (Alchem, Wroclaw, Poland) for 30 s. Samples were examined on simple Olympus microscopy (Japan). Stained cell numbers were determined by counting 100 cells in 3 randomly selected fields. The counting was performed by two independent investigators. The result was judged to be positive if staining was observed in more than 5% of cells. The intensity of immunohistochemical staining was evaluated as (–) negative (no reaction), (+) weak, (++) moderate, and (+++) strong. All experiments were repeated three times.

### 2.9. Statistical Analysis

The experiments were performed in triplicates. The obtained and calculated data was expressed as mean ± SD and analyzed by one-way ANOVA (in GraphPad Prism 7.05), with *p* < 0.05 or *p* < 0.005 considered as a statistically significant.

## 3. Results

### 3.1. Cell Viability, Confluency and Volume

**Figure 1:**
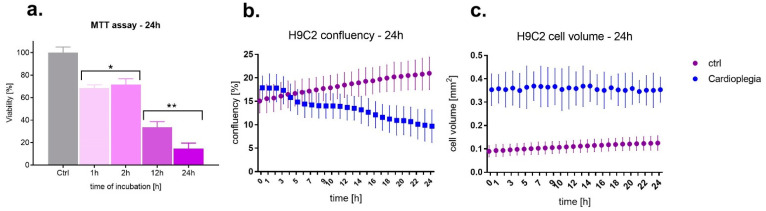
The effect of St. Thomas 2 based cardioplegia (ST2C) on H9C2 cells (**a**) viability measured by MTT assay; (**b**) confluency and (**c**) cell volume after 24 h exposure. The viability results presented as the mean ± SD. One-way ANOVA analysis: * *p* < 0.05, ** *p* > 0.005.

The effect of St. Thomas 2 based cardioplegia (ST2C) was validated using the analysis of the viability, confluency, and the H9C2 cells volume. The results are shown in Figure 1. It was noticed that short times of exposure did not affect cell viability and confluency. In the case of viability, the longest time was 2 h Figure 1a, when cells’ viability maintained above 50% of the untreated control. The analysis of the confluency indicated 4 h as a cut-off time, whereafter the confluency began to fall (Figure 1b). The cell volume analysis indicated that cells exposed to cardioplegic solution increased their volume, swelling, which was maintained on the same during 24 h (Figure 1c). We can observe the difference in the initial cell volume, which can be related to the experimental procedure protocol. Cells were exposed to ST2C a minimum 10–15 before measurements. Thus, this short pre-incubation time could cause cell swelling, which is maintained for 24 h. In the case of control cells, we observe normal cell volume, which is also maintained during the same time.

### 3.2. Cardioplegia Effects on Myocytes’ Morphology

**Figure 2:**
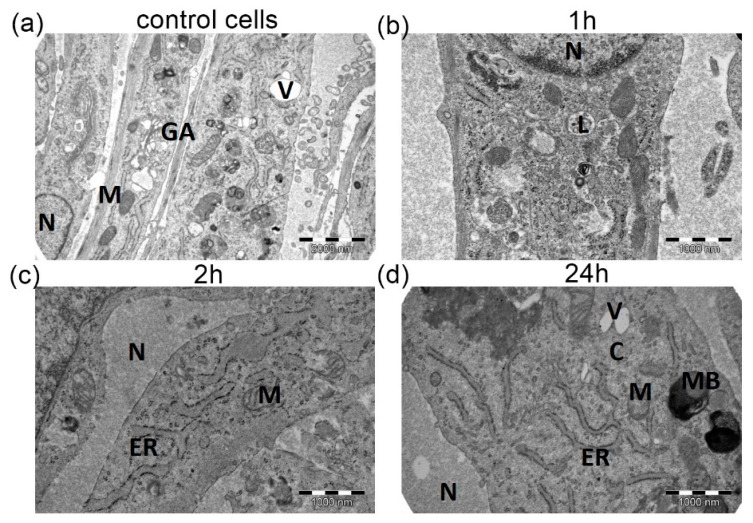
The transmission electron micrography of H9C2 cells: (**a**) untreated control; after (**b**) 1 h; (**c**) 2h and (**d**) 24 h with ST2C based cardioplegia. C: cytoplasm M: mitochondria, N: nucleus, ER: endoplasmic reticulum, GA—Golgi apparatus; MB—myeline bodies; V—vacuoles; L—lysosomes.

The changes of the ultrastructure in H9C2 cells exposed to cold cardioplegia were time-dependent. Figure 2 shows TEM visualization after the exposure to St. Thomas 2 based cardioplegia (ST2C). After 1h incubation with cold cardioplegia, insignificant pathological changes of cardiomyocytes mitochondria were observed in comparison to control untreated cells. (Figure 2b). H9C2 cells exposed for 2 h normal mitochondria. The cristae of the mitochondria were slightly swollen, which seems compatible with the cell volume results. However, the nucleus revealed normal, unaffected size and shape. Also, we observed slightly swollen endoplasmic reticulum (Figure 2c). After 24 h incubation with cardioplegia, vacuoles and myelin bodies were formed, and some contained electron dense material (Figure 2d). Endoplasmic reticulum and mitochondria revealed normal morphology as compared to unexposed control cells.

**Figure 3:**
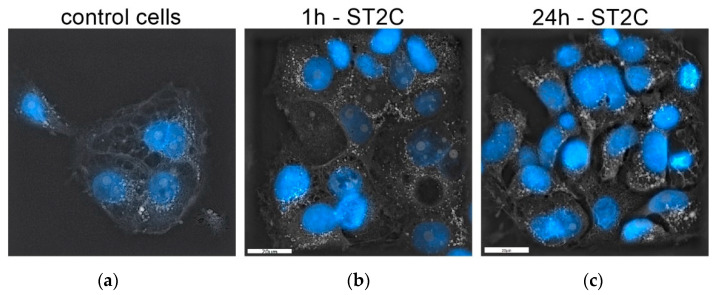
The holotomographic microscopy of H9C2 cells: (**a**) untreated control; after (**b**) 1 h exposure to ST2C; and (**c**) 24 h exposure to ST2C. Blue color–nuclei stained by DAPI marker.

In Figure 3, 3D visualization of H9C2 cells is presented. There can be seen not disturbed morphology. Only longer time of the incubation provoked an increased vacuolization, similarly to TEM studies.

**Figure 4:**
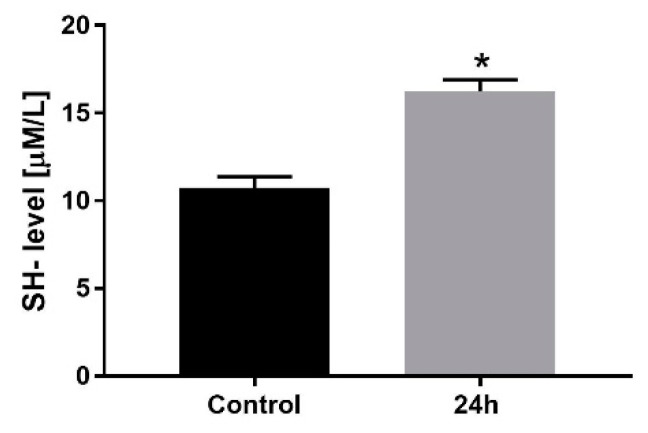
The level of sulfhydryl groups (SH-) as a protein damage marker, in rat myoblasts after 24 h incubation with ST2C based cardioplegia. The data are presented as µM/L protein and expressed as the means ± SD. * *p* < 0.05.

In the next step, the level of thiol groups was measured after 24 h and is shown in Figure 4. The longest time of the exposition was selected, as it was the most harmful for H9C2 cells. There was observed cold crystalloid cardioplegia significantly increased the level of SH- groups, which can be associated with the increased volume and cells’ swelling.

Table 1 and Table 2 demonstrate results for proliferation markers, i.e., PCNA and Ki67 [14]. The obtained results showed an increased expression of both markers after 24 h exposure to ST2C, which was visualized by the immunostained reaction.

The analysis of PCNA indicated an increased number of cells which underwent stained reaction after 24 h post ST2C exposure. In the case of Ki67, we could detect medium stained reaction in all cases in the almost of 100% of cells. However, the 24 h revealed the most intense immunostained reaction.

## 4. Discussion

Cells’ condition and their viability and proliferation ability are of high importance in the case of cardiac surgery; thus, cardioplegic solutions are widely used [2,15]. In this study, the effect of St. Thomas 2 based cardioplegia (ST2C) was validated on the viability, confluency, and the H9C2 cells volume. Interestingly, Stephenson et al. [16] observed myocyte swelling just after the exposure to cold St. Thomas’ solution for up to 30 min. In our study, longer observations were performed. After 2 h exposure occurred as the optimal time for St. Thomas modified cardioplegia, however, we indicated that it should not exceed 4 h, according to the confluency measurements. We conclude that cells conditions are still satisfactory until cells can maintain confluency on the control cells level. and the obtained results indicated that cells sustain in good conditions up to 4 h. The available studies highlighted that cell swelling occurring after the exposition to cardioplegia is avoided by normothermic infusion [16,17].

In the case of cells conditions, protein damage level is a direct marker of protection. The reduction of thiols is also a marker of oxidative stress and also morbidity and mortality [18]. The available data clarify that thiol groups play an important role in electron transport, oxidative phosphorylation, and also swelling and contraction [19]. We have demonstrated that ST2C based cardioplegia can act as an agent for metabolic and antioxidant protection in neonatal myoblasts.

Protection during cardiac surgical procedures is one of the essential elements and is determined by the damage of ventricular contraction and prevention against increased myocardial enzymes [20]. It was previously demonstrated that cold crystalloid cardioplegia can be successfully used in cardiac surgery and recovery [5,21]. In this study we used St. Thomas No. 2 based cardioplegia, which is commonly used solution of crystalloid cardioplegia in cardiac surgery. ST2C should be delivered repeatedly at short intervals during the surgery [13]. It was demonstrated that molecular mechanism of cold crystalloid cardioplegia is related to the aquaporins (AQPs), which are integral membrane proteins and act as water channels. The expression of these specific proteins increases under ischemia conditions, in particular AQP4 and AQP7. It was also found that AQP7 deficiency, protected myocardial cells [22]. The other studies demonstrated that cardioplegic arrest is also strictly related to the altered calcium and potassium ions circuits, and finally, prolonged membrane depolarization [6]. In this study, neonatal rat myoblasts were used as a model of extremely sensitive cells, and ST2C based cardioplegia was used in this in vitro model to estimate cell conditions. Numerous studies indicate that cardioplegia causes cell swelling, which was also observed in our study. However, we have determined the maximum incubation time with ST2C to maintain neonatal myocytes in good conditions and proliferation abilities [17,23]. Furthermore, Fedosova et al. demonstrated that blood and crystalloid cardioplegia can be effectively used in patients with a long cross-clamp time [24]. Our results also indicate that cold crystalloid cardioplegia can have protective properties to maintain cells’ conditions for up to 4 h.

In vitro studies cannot be easily and directly transferred into clinical practice. Our laboratory model does not refer to the reperfusion injury after the period of ischemia. Cardioplegic protection includes all cells of the myocardium and coronary vessels. Our research relates the most important but only one aspect of protection strategy-cardiomyocytes themselves. The future direction of our research would be improving the in vitro model of ischemia/reperfusion injury and the application of different cells derived from endothelium.

## 5. Conclusions

The protection of cardiac muscle during surgical procedures is critical. Our study of in vitro model demonstrated that ST2 based cardioplegia can protect and maintain H9C2 rat cardiomyocytes by increasing protein disulfides and proliferation markers (PCNA and Ki67), without significant morphological changes. However, the time of protection is limited to 4 h in the case of sensitive neonatal myoblasts. Ultrastructural and 3D visualizations demonstrated no significant alternation in the exposed cells. The obtained results indicate that ST2C can be used to protect the myocardial cells, and it is a fairly inexpensive and simple solution. Our studies confirmed protein protection, and that myocardial cell morphology was not affected after exposure to ST2C. Thus, the use of St. Thomas no. 2 modified cardioplegia seems to be promising in protecting cardiac muscle cells, particularly neonatal myoblasts.

## Figures and Tables

**Table 1:** Analysis of PCNA expression in H9C2 cells after incubation with ST2C based cardioplegia (* *p* < 0.05).

		Intensity of ABC Reaction	[%] of Stained Cells
Control		-	-
Incubation time	1 h	-	-
	2 h	-/+	5 ± 2
	24 h	++/+++	90 ± 9 *

No reaction (-); weak reaction (+), moderate reaction (++), strong reaction (+++)

**Table 2:** Analysis of Ki67 expression in H9C2 cells after incubation with ST2C based cardioplegia (* *p* < 0.05).

		Intensity of ABC Reaction	[%] of Stained Cells
Control		++	99 ± 4
Incubation time	1 h	++	98 ± 3
	2 h	++	87 ± 6
	24 h	++/+++	95 ± 5 *

No reaction (-); weak reaction (+), moderate reaction (++), strong reaction (+++)

## Data Availability

Not applicable.
